# Biophysical Analysis of a Minimalistic Kidney Model Expressing SGLT1 Reveals Crosstalk between Luminal and Lateral Membranes and a Plausible Mechanism of Isosmotic Transport

**DOI:** 10.3390/biom14080889

**Published:** 2024-07-23

**Authors:** Erik Hviid Larsen, Jens Nørkær Sørensen

**Affiliations:** 1 Department of Biology, University of Copenhagen, DK-2100 Copenhagen, Denmark; 2 Department of Wind Energy, Technical University of Denmark, DK-2800 Lyngby, Denmark; jnso@dtu.dk

**Keywords:** kidney proximal tubule, SGLT1, glucose uptake by the S3 segment, water transport by SGLT1, crosstalk between apical SGLT1 and lateral Na/K pump, isosmotic fluid absorption, glucose clearing, mathematical modeling

## Abstract

We extended our model of the S1 tubular segment to address the mechanisms by which SGLT1 interacts with lateral Na/K pumps and tight junctional complexes to generate isosmotic fluid reabsorption via tubular segment S3. The strategy applied allowed for simulation of laboratory experiments. Reproducing known experimental results constrained the range of acceptable model outputs and contributed to minimizing the free parameter space. (1) In experimental conditions, published Na and K concentrations of proximal kidney cells were found to deviate substantially from their normal physiological levels. Analysis of the mechanisms involved suggested insufficient oxygen supply as the cause and, indirectly, that a main function of the Na/H exchanger (NHE3) is to extrude protons stemming from mitochondrial energy metabolism. (2) The water path from the lumen to the peritubular space passed through aquaporins on the cell membrane and claudin-2 at paracellular tight junctions, with an additional contribution to water transport by the coupling of 1 glucose:2 Na:400 H_2_O in SGLT1. (3) A Na-uptake component passed through paracellular junctions via solvent drag in Na- and water-permeable claudin-2, thus bypassing the Na/K pump, in agreement with the findings of early studies. (4) Electrical crosstalk between apical rheogenic SGLT1 and lateral rheogenic Na/K pumps resulted in tight coupling of luminal glucose uptake and transepithelial water flow. (5) Isosmotic transport was achieved by Na-mediated ion recirculation at the peritubular membrane.

## 1. Introduction

Kidney proximal tubules mediate essential restorative functions from glomeruli filtrates. Approximately two-thirds of filtered sodium and water are reabsorbed iso-osmotically, and over 99% of filtered glucose is reabsorbed by the end of the straight S3 tubular segment in humans. There is a vast body of literature on the intraepithelial pathways involved in this process, but the mechanisms by which this restoration is accomplished remain to be elucidated. This is the aim of our investigation. An investigation of mechanisms of such complexity involving the entangled operation of multiple cellular and intercellular components requires a modeling approach. The absorption of glucose by the kidney proximal tubule is accompanied by the uptake of Na and water. The luminal membrane proteins SGLT1 and SGLT2 constitute the first step of transepithelial glucose uptake with a stoichiometry of 2 Na:1 glucose and 1 Na:1 glucose, respectively. In humans, 180 L/day is produced by glomerular filtration, and the proximal tubule reabsorbs 120 L/day together with Na. In the kidney, the cotransport of Na and glucose [1] as well as the cotransport of glucose and water are well established at the membrane level [2,3,4]. Our previous study [5,6] predicted that water uptake in proximal convoluted tubules is tightly associated with glucose stimulation of SGLT2 in agreement with experimental observations [7,8]. The aim of the present study was to analyze two major functions of the proximal straight tubule: first, the principles governing the quantitative relationship between glucose absorption and water uptake, with emphasis on the relationship between osmotic water transport and water uptake via the SGLT1 transporter under physiological conditions; second, the capacity of straight tubules to absorb luminal fluid isosmotically.

## 2. Functional Organization of Proximal Straight Tubules

**Figure 1:**
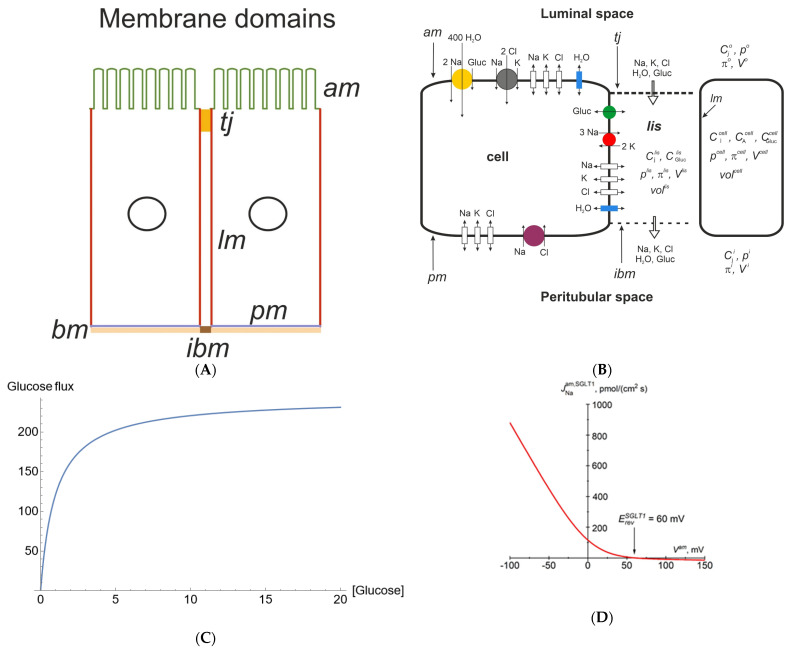
(**A**) Membrane organization of the kidney tubule epithelium. The apical (*am*) and lateral (*lm*) cell membranes have similar relatively large areas compared with the contra-luminal (peritubular) cell membrane (*pm*) contacting the basement membrane (*bm*). Tight junctions (*tj*) provide entrance to the lateral intercellular space (*lis*). The so-called ‘basal infoldings’ are continuous with the lateral membranes; the interspace basement membrane (*ibm*) providing an exit from *lis* is approximately 10% of the area of the entire basement membrane. Sodium pumps are confined to the lateral membranes and to the complex of lateral membrane infoldings at the cell base. Thus, lateral and peritubular membranes represent different functional domains, implying that sodium ions transported into the cell through the *am* are actively transported into the *lis* in order to exit the epithelium through the *ibm*. It follows that the so-called ‘basolateral’ osmotic permeability conferred by AQP1 is confined to lateral membranes, with the lateral intercellular space constituting a compartment of its own. (**B**) Functional membrane organization of the proximal tubule S3 segment. SGLT1 (yellow) couples 1 glucose with 2 Na and 400 H_2_O. The driving force for glucose is given by the transmembrane concentration differences of sodium and glucose and the apical membrane potential, according to Equation (3b). Together with paracellular solvent drag, the apical 1Na–2Cl–1K cotransporter ensures the reabsorption of filtered K. By being water-permeable, the junctional ion and glucose pathways play important roles in preventing the loss of glucose via urine. In modeling isosmotic transport, the serosal Na–Cl cotransporter was activated iteratively to achieve overall osmotic equilibrium between the peritubular and reabsorbed fluid. (**C**) ‘Saturation’ of the glucose flux across the apical membrane (pmol/cm^2^/s) with the luminal glucose concentration (mM), which, in our mathematical analysis, was due to glucose concentration-dependent SGLT1 permeability according to Equation (3a) [16]. (**D**) Instantaneous relationship between the Na flux carried by SGLT1 and apical membrane potential according to Equation (3b). The constant electrical field in the membrane and the fixed stoichiometry of 2 Na–1 glucose predict strong inward rectification of SGLT1 fluxes. The reversal potential, $E_{rev}^{SGLT1}$ , is +60 mV at physiological concentrations and 37 °C, i.e., ~125 mV above the apical membrane potential of approximately −65 mV.

The biophysical model comprises four well-stirred compartments: outer (lumen, *o*) and peritubular (*ps*), both of infinite volume, cell (*c*), and lateral intercellular space (*lis*) confined by five membranes: the apical (*am*), lateral (*lm*), tight junction (*tj*) and intercellular basement membrane (*ibm*) (Figure 1A). It should be noted that apical and lateral cell membranes have similar and relatively large areas compared with the peritubular (contra-luminal) membrane contacting the basement membrane [9]. Because they are continuous with the lateral membrane, the so-called ‘basal infoldings’ are not derived from the contra-luminal membrane. It follows that the interspace basement membrane (*ibm*), which constitutes approximately 10% of the area of the entire basement membrane [10], provides the exit path from the lateral intercellular space (*lis*). Sodium pumps are confined to lateral membranes and to the complex of lateral membrane infoldings at the cell base [11] (Figure 1B). Thus, lateral and peritubular membranes represent different functional domains, implying that sodium ions transported into the cell through *am* are actively transported into the lateral intercellular space (*lis*) in order to exit the epithelium through *ibm*. It follows that the so-called ‘basolateral’ osmotic permeability conferred by AQP1 [12,13] is confined to lateral membranes, with the lateral intercellular space constituting a compartment of its own. The membrane organization of our minimalistic model of proximal straight tubules is depicted in Figure 1B and is described in detail below. The primary unknowns include the cellular and paracellular concentrations of Na, K, Cl, glucose and nondiffusible intracellular anions; hydrostatic pressure in the cell and lateral intercellular space; and electric potential in the cell, *lis*, and luminal space with the electric potential of the peritubular space, $\psi^{ps}\equiv 0$ .

The electroneutral NHE3 sodium ion/proton antiporter of the apical membrane discovered by Murer et al. [14] and studied in detail by Boron and coworkers, e.g., [15] mediates transcellular uptake of bicarbonate. Below, we discuss evidence for another function of the NEH3 transporter, which is to export the free protons generated during oxidative metabolism by physiologically active tubule cells. In the absence of experimental data connecting NHE3 to glucose transport, its inclusion in the model at this stage was considered premature. With our strategy of using known experimental results to constrain model outcomes and minimize the free parameter space, its inclusion would be justified if the assembly of the current model components failed to account for glucose clearance or as part of a wider exploration of unanticipated side effects.

## 3. Mathematical Description

Definitions and symbols of variables and constants are given in Appendix A.

### 3.1. Cellular Solute Flux Equations

Electrodiffusion fluxes, including the flux in the apical Na channel [17,18,19], are handled by the GHK constant field-equation with the associated integral conductance equation [20,21]:

(1a) $$J_{j}=\left(\frac{z_{j}FV}{RT}P_{j}\right)\frac{C_{j}^{\left(I\right)}\exp[z_{j}FV/(RT)]-C_{j}^{\left(II\right)}}{\exp[z_{j}FV/(RT)]-1}$$

(1b) $$G_{j}=\left(\frac{{\left(z_{j}F\right)}^{2}V}{RT}P_{j}\right)\frac{C_{j}^{I}\exp\left[z_{j}FV\right]-C_{j}^{II}}{\left(\exp\left[z_{j}FV/RT\right]-1\right)\left(V-E_{j}\right)}$$

*P_j_* is the ion permeability and *V* is the potential difference across the membrane. I = *lumen* and II = *cell* for the apical membrane, I = *cell* and II = *lis* for the lateral membrane, and I = *cell* and II = *peritubular space* for the serosal membrane. As previously discussed, the principal importance of uphill K absorption by the proximal tubule is that the potassium concentration of the absorbate is close to that of the extracellular fluid. Potassium absorption is mediated both by paracellular solvent drag and by secondary active transport, the latter of which is driven by the prevailing inwardly directed electrochemical sodium gradient [22]. The precise mechanism of uphill K uptake is unknown. In our model [5], potassium absorption is handled by an hypothetical 1 Na, 1 K, 2 Cl cotransporter,

(2) $$J_{j}^{NaK2Cl,am}=r\cdot K^{NaK2Cl,am}\left[C_{Na}^{\left(I\right)}\cdot C_{K}^{\left(I\right)}\cdot {\left(C_{Cl}^{\left(I\right)}\right)}^{2}-C_{Na}^{\left(II\right)}\cdot C_{K}^{\left(II\right)}\cdot {\left(C_{Cl}^{\left(II\right)}\right)}^{2}\right]$$

where *r* = 1 for Na and K and *r* = 2 for Cl. The application of Equation (2) has the additional advantage of enabling apical secondary active Cl uptake to be electroneutral, which is consistent with cellular Cl accumulation via Na-dependent transport in *Necturus* [23,24,25] and rats [26]. These two early studies on vertebrate kidneys are important for our argumentaion as they indicate that the flux of C1 from the lumen of the proximal tubule into the cells is mediated by and dependent on the presence of luminal Na. Furthermore, the application of current clamping indicated that intracellular chloride activity was insensitive to alterations in luminal membrane potential, suggesting that Cl entry was electrically neutral. It is of relevance here to also mention that the recovery of luminal K according to Equation (2) is associated with relatively very small currents (see below) of little significance for the overall bioelectrical properties of the tubular epithelium.

The proximal tubule reabsorbs most of the glucose in the glomerular filtrate [27]. In the tubule’s S3 segments, glucose is coupled to Na uptake via SGLT1 at a ratio of 2 Na:1 glucose [1]. SGLT1 also functions as a low-conductance water channel that stoichiometrically couples Na, sugar and water, with 1 glucose: 2 Na: 400 H_2_O [28,29] (Figure 1B). The driving forces for glucose uptake across the luminal membrane are the transmembrane glucose concentration gradient, the transmembrane Na concentration gradient, and the apical membrane potential, *V^am^*. Typically, for thin membranes, the electric field is assumed to be constant [30]. To simplify the integration, we assumed that one glucose and two Na molecules pass through the membrane ‘in unity as a divalent particle’, in accordance with a recent model of SGLT1 transport [31]. In our handling of the ‘saturation kinetics’ of glucose uptake from tubular fluid, we introduced a concentration-dependent SGLT1 permeability defined by [16],

(3a) $$P^{SGLT1}=P^{SGLT1,\max}[K^{SGLT1}/(C_{Gluc}^{o}+K^{SGLT1})]$$

Thus, the following three instantaneously coupled flux equations apply:

(3b) $$\begin{array}{l} J_{Gluc}^{SGLT1}=\frac{P^{SGLT1,\max}K^{SGLT1}}{\left(C_{Gluc}^{o}+K^{SGLT1}\right)}\frac{2FV^{am}}{RT}\frac{C_{Gluc}^{o}{\left(C_{Na}^{o}\right)}^{2}-C_{Gluc}^{c}{\left(C_{Na}^{c}\right)}^{2}\exp\{2FV^{am}/(RT)\}}{\exp\{2FV^{am}/(RT)\}-1}\\ J_{Na}^{SGLT1}=2\times J_{Gluc}^{SGLT1}\\ J_{H_{2}O}^{SGLT1}=400\times J_{Gluc}^{SGLT1} \end{array}$$

which assumes that 2 Na and 400 water molecules are transported for each turnover of the glucose transporter [28,32]. Figure 1C shows the concentration dependence of luminal glucose uptake at a constant apical membrane potential indicating ‘saturation kinetics’ in agreement with observations [1]. Voltage-clamp studies of cloned SGLT1 [33] revealed a near-linear dependence of SGLT1 currents within the voltage range of −40 to −100 mV [31], which is reproduced by the above Equation (3b) (Figure 1D). Independent of the shape of the current–voltage relationship, the reversal potential, $E_{rev}^{am,SGLT1}$ , is that of the Na flux carried by SGLT1,

(3c) $$E_{rev}^{am,SGLT1}=\frac{RT}{2F}\ln\frac{C_{Gluc}^{o}{\left(C_{Na}^{o}\right)}^{2}}{C_{Gluc}^{c}{\left(C_{Na}^{c}\right)}^{2}}$$

In the physiological range of membrane potentials, the integral conductance of SGLT1 is

(3d) $$G_{Gluc,Na}^{SGLT1}=\frac{2FV^{am}P_{Gluc,Na}^{SGLT1,\max}}{RT}\frac{K_{Gluc,Na}^{SGLT1}}{\left(C_{Gluc}^{o}+K_{Gluc,Na}^{SGLT1}\right)}\frac{C_{Gluc}^{o}{\left(C_{Na}^{o}\right)}^{2}-C_{Gluc}^{c}{\left(C_{Na}^{c}\right)}^{2}\exp\{2FV^{am}/(RT)\}}{\left(\exp\{2FV^{am}/(RT)\}-1)(V^{am}-E_{rev}^{am,SGLT1}\right)}$$

The exit flux of glucose via GLUT1 [34] from cell to *lis* is handled by Stein’s equation for a symmetrical, saturating carrier [35],

(4) $$J_{Gluc}^{lm}=J_{Gluc}^{lm,\max}\frac{K_{Gluc}^{lm}(C_{Gluc}^{c}-C_{Gluc}^{lis})}{\left(K_{Gluc}^{lm}+C_{Gluc}^{c})(K_{Gluc}^{lm}+C_{Gluc}^{lis}\right)}$$

The sodium pump is expressed exclusively in the lateral membrane [11]. The rate of active cation fluxes is a saturating function of the concentration of Na in the cell, $C_{Na}^{c}$ , and the concentration of K in the lateral intercellular space, $C_{K}^{lis}$ [36]. The rate of pumping of the two cations across the lateral membrane (*lm*) with a stoichiometry of 3Na/2K is also a function of the electric work performed while moving one charge across the membrane per pump cycle [37], which is dependent on the membrane potential [38]. Equation (5) below fulfills these requirements [5,39].

(5a) $$\begin{array}{l} J_{Na}^{lm,pump}=P_{Na,K}^{lm,pump}{\left(\frac{C_{Na}^{c}}{K_{Na}^{lm,pump}+C_{Na}^{c}}\right)}^{3}{\left(\frac{C_{K}^{lis}}{K_{K}^{lm,pump}+C_{K}^{lis}}\right)}^{2}\left[V^{lm}+E^{pump}\right]\\ J_{K}^{lm,pump}=-\frac{2}{3}J_{Na}^{lm,pump}\\ V_{m}^{lm}=\psi^{cell}-\psi^{lis}\\ I_{Na}^{lm,pump}=\frac{F\cdot P_{Na,K}^{lm,pump}}{3}{\left(\frac{C_{Na}^{c}}{K_{Na}^{lm,pump}+C_{Na}^{c}}\right)}^{3}{\left(\frac{C_{K}^{lis}}{K_{K}^{lm,pump}+C_{K}^{lis}}\right)}^{2}\left[V^{lm}+E^{pump}\right] \end{array}$$

where *F* is the Faraday constant. Introducing the pump conductance, $G^{lm,pump}$ we have:


$$I_{Na}^{lm,pump}=G^{lm,pump}[V^{lm}+E^{pump}]$$

(5b) $$G^{lm,pump}=\frac{F\cdot P_{Na,K}^{lm,pump}}{3}{\left(\frac{C_{Na}^{c}}{K_{Na}^{lm,pump}+C_{Na}^{c}}\right)}^{3}{\left(\frac{C_{K}^{lis}}{K_{K}^{lm,pump}+C_{K}^{lis}}\right)}^{2}$$

The dimension of $P_{Na,K}^{lm,pump}$ is mol∙s^−1^∙V^−1^ and that of $G^{lm,pump}$ is Siemens, both per unit area of apical membrane. $K_{Na}^{lm,pump}$ = 3.4 mM and $K_{K}^{lm,pump}$ = 0.75 mM [40]. According to Ussing [41], *E^pump^* is the *electromotive force of the pump* given by the change in free energy of ATP hydrolysis in the vicinity of the pump, which is numerically equal to the reversal potential of the pump current, $V_{rev}^{pump}$ . Assuming a change in the free energy of ATP hydrolysis of $\Delta G_{ATP}$ = −58 kJ/mole [42,43,44,45], stoichiometry of 3 Na–1 ATP, mitochondria situated near pump sites [46], and well-aerated tubules, the reversal potential of the pump $V_{rev}^{pump}$ = (−58 × 10^3^/3*F*) × 10^3^ = −200 mV. This agrees with the upper limit value obtained in experiments on isolated frog skin epithelium [41]. In the physiological range of membrane potentials, Equation (5a) generates pump currents that are near-linearly dependent on *V^lm^*, which is in agreement with experiments [38].

### 3.2. Paracellular Flux Equations

We treat paracellular solvent drag on small diffusible ions using Hertz’s equation [20,47] applied to a membrane with a nonzero reflection coefficient, σ*_j_*, of ion j [48,49],

(6) $$J_{j}=\left(\frac{z_{j}FV}{RT}P_{j}+J_{V}(1-\sigma_{j})\right)\frac{C_{j}^{\left(I\right)}\exp[z_{j}FV/(RT)]\exp[J_{V}(1-\sigma_{j})/P_{j}]-C_{j}^{\left(II\right)}}{\exp[z_{j}FV/(RT)]\exp[J_{V}(1-\sigma_{j})/P_{j}]-1}$$

For a tight junction membrane, I = *o*, while II = *lis*. For an interspace basement membrane, I = *lis* and II = *ps*. Equation (6) assumes a symmetric pore with a reflection coefficient ( $\sigma_{j}$ ) and partition coefficient ( β ) related by $\sigma_{j}=1\;–\beta$ [50]. *J_V_* is the volume flow from compartment I (lumen or *lis*) to II (*lis* or peritubular space). Equation (6) applies to Na and K passing through paracellular tight junctions through the water-permeable claudin-2 [51,52], and is applied for solutes passing through non-specified pathways of *ibm*. Claudin-17/10a are permeable to small anions but not to water [51,53]; therefore, the junctional Cl flux is calculated by the formula for simple electrodiffusion (Equation (1)). Solvent drag on sucrose and other electroneutral molecules in the kidney proximal tubule [54,55] makes it plausible that electroneutral glucose is also subjected to paracellular transport by frictional interactions with water. This flux may correspond with the component of glucose uptake in the proximal tubule designated ‘moderate glucose leak’ [56]. The paracellular glucose fluxes across a tight junction (*tj*) and interspace basement membrane (*ibm*) are computed by [48].

(7a) $$J_{Gluc}^{tj}=J_{V}^{tj}(1-\sigma_{Gluc}^{tj})\frac{C_{Gluc}^{o}\exp[J_{V}^{tj}(1-\sigma_{Gluc}^{tj})/P_{Gluc}^{tj}]-C_{Gluc}^{lis}}{\exp[J_{V}^{tj}(1-\sigma_{Gluc}^{tj})/P_{Gluc}^{tj}]-1}$$

(7b) $$J_{Gluc}^{ibm}=J_{V}^{ibm}(1-\sigma_{Gluc}^{ibm})\frac{C_{Gluc}^{lis}\exp[J_{V}^{ibm}(1-\sigma_{Gluc}^{ibm})/P_{Gluc}^{lis}]-C_{Gluc}^{ps}}{\exp[J_{V}^{ibm}(1-\sigma_{Gluc}^{ibm})/P_{Gluc}^{lis}]-1}$$

### 3.3. Water Flow via Aquaporins

In agreement with the cloned aquaporins of proximal tubules [12], we assume a reflection coefficient of unity for water flow through cell membranes. With *i* denoting the individual element or molecule, the equations for the respective volume flows per unit of area of the apical membrane are

(8a) $$J_{V}^{am}=L_{p}^{am}\left\{RT\left(\sum C_{i}^{c}-\sum C_{i}^{o}\right)+(p^{o}-p^{c})\right\}$$

(8b) $$J_{V}^{lm}=L_{p}^{lm}\left\{RT\left(\sum C_{i}^{lis}-\sum C_{i}^{c}\right)+(p^{c}-p^{lis})\right\}$$

(8c) $$J_{V}^{pm}=L_{p}^{pm}\left\{RT\left(\sum C_{i}^{ps}-\sum C_{i}^{c}\right)+(p^{c}-p^{ps})\right\}$$

As the osmotic permeability of the individual SGLT1 channel is no more than approximately 1% of that of a single aquaporin molecule (AQP1) independent of the presence of glucose [29], we assume that the small *osmotic* water flow through SGLT1 is included in Equation (8a). The equations for water flow through membranes *tj* and *ibm*, delimiting the lateral intercellular space from luminal and peritubular solutions, respectively, include reflection coefficients,

(9a) $$J_{V}^{tj}=L_{p}^{tj}\left\{RT\left(\sum \sigma_{i}^{tj}(C_{i}^{lis}-C_{i}^{o})\right)+(p^{o}-p^{lis})\right\}$$

(9b) $$J_{V}^{ibm}=L_{p}^{ibm}\left\{RT\left(\sum \sigma_{i}^{ibm}(C_{i}^{ps}-C_{i}^{lis})\right)+(p^{lis}-p^{ps})\right\}$$

The reflection coefficients of tight junctions are obtained from the literature, $\sigma_{Na}^{tj}=\sigma_{K}^{tj}=0.7$ and $\sigma_{Cl}^{tj}=0.45$ [57], while for large electroneutral glucose, we assume that $\sigma_{Gluc}^{tj}=0.8$ . The computations to be presented are not sensitive to this choice as long as $\sigma_{Gluc}^{tj}>>\sigma_{Gluc}^{ibm}$ . Generally, for fluid absorption, the reflection coefficients of *ibm* must be smaller than those of tight junctions; here, we assume that $\sigma_{i}^{ibm}$ = 10^−4^. In the literature, the hydraulic conductance *L_p_* is translated to the osmotic permeability, *P_f_*. With the molar volume of water indicated by ${\bar{V}}_{W}$ , *L_p_* and *P_f_* are related by [50],

(10) $$P_{f}=\frac{RTL_{p}}{{\bar{V}}_{W}}$$

### 3.4. Electroneutrality

With the mean valence of nondiffusible anions in the cell denoted *z_A_*, the electroneutrality requirements are:

(11) $$C_{A}^{c}=(C_{Na}^{c}+C_{K}^{c}-C_{Cl}^{c})/z_{A}$$

(12) $$C_{Cl}^{lis}=C_{Na}^{lis}+C_{K}^{lis}$$

If *I^clamp^* is the transepithelial clamping current and *I_j_* is the current carried by *j* through the membrane indicated by superscript (*j* = Na, K, or Cl), the mathematical solution would have to obey the following requirement:

(13) $$I^{clamp}=I_{Na}^{am}+I_{Na}^{tj}+I_{K}^{am}+I_{K}^{tj}+I_{Cl}^{am}+I_{Cl}^{tj}$$

Here, *I^clamp^* = 0 defines the mathematical solution containing the transepithelial potential difference *V^trans^*.

### 3.5. Hydrostatic Pressure and Intraepithelial Volumes

To obtain the hydrostatic pressures of the cell and *lis*, we apply the following compliance model [5]:

(14) $$p^{c}=(\mu^{am}p^{o}+\mu^{lm}p^{lis}+\mu^{pm}p^{ps})/(\mu^{am}+\mu^{lm}+\mu^{pm})$$

in which *µ* is the compliance factor of the membrane indicated. With *lis* volume in the absence of fluid flow indicated by *Volis^lis, ref^* the cell volume is calculated by [5],

(15) $$Vol^{lis}=Vol^{lis,ref}[1+\mu^{lm}(p^{lis}-p^{c})]$$

We denote the number of cells per unit of area of the apical membrane and the amount of nondiffusible anions per cell *D^c^* and *M_A_*, respectively. Hence, the volume of the functional syncytium is:

(16) $$Vol^{c}=D^{c}M_{A}/C_{A}^{c}$$

Here $C_{A}^{c}$ is the dependent variable.

### 3.6. Electrical-Circuit Analysis and Sign Conventions

Because the transepithelial potential difference is a function of current flow in cellular and paracellular pathways, for a given set of independent variables, *V^trans^* is computed after the solution to the above set of equations is obtained. Our method makes use of the resistance of each of the five membranes of the equivalent bridge circuit [5] *R^m^* (*m* = 1–5), obtained from the summation of the chord conductance of the individual ion pathways, i.e., Equation (1b) for ion channels, Equation (3c) for SGLT1, and Equation (5b) for the Na/K pump. To simulate a step change in the transepithelial current, Δ*I*^trans^ and Kirchhoff’s rules are applied to set up five simultaneous linear equations:

(17a) $$\begin{array}{l} I_{1}R_{1}+I_{3}R_{3}-I_{4}R_{4}=0 \end{array}$$

(17b) $$\begin{array}{l} I_{3}R_{3}+I_{5}R_{5}-I_{2}R_{2}=0 \end{array}$$

(17c) $$\begin{array}{l} I_{2}+I_{5}-\Delta I^{trans}=0 \end{array}$$

(17d) $$\begin{array}{l} I_{1}-I_{2}-I_{3}=0 \end{array}$$

(17e) $$\begin{array}{l} I_{4}-I_{3}-I_{5}=0 \end{array}$$

The currents *I_n_* (*n* = 1–5) flowing through the five resistors were obtained through the ‘Solve routine’ of Mathematica©:

(18a) $$I_{1}=-\frac{-(R_{2}R_{4}+R_{3}R_{4}+R_{3}R_{5}+R_{4}R_{5})}{R_{1}R_{2}+R_{1}R_{3}+R_{2}R_{3}+R_{2}R_{4}+R_{3}R_{4}+R_{1}R_{5}+R_{3}R_{5}+R_{4}R_{5}}\Delta I^{trans}\;$$

(18b) $$I_{2}=\Delta I^{trans}+\frac{\left(R_{1}R_{3}+R_{2}(R_{1}+R_{3}+R_{4})\right)}{-R_{1}R_{3}-R_{1}R_{4}-R_{2}(R_{1}+R_{3}+R_{4})-R_{5}(R_{1}+R_{3}+R_{4})}\Delta I^{trans}$$

(18c) $$I_{3}=-\frac{\left(R_{2}R_{4}+R_{1}R_{5}\right)}{R_{1}R_{2}+R_{1}R_{3}+R_{2}R_{3}+R_{2}R_{4}+R_{3}R_{4}+R_{1}R_{5}+R_{3}R_{5}+R_{4}R_{5}}\Delta I^{trans}$$

(18d) $$I_{4}=-\frac{-(R_{1}R_{2}-R_{1}R_{3}-R_{2}R_{3}-R_{1}R_{5})}{R_{1}R_{2}+R_{1}R_{3}+R_{2}R_{3}+R_{2}R_{4}+R_{3}R_{4}+R_{1}R_{5}+R_{3}R_{5}}\Delta I^{trans}$$

(18e) $$I_{5}=-\frac{\left(R_{1}R_{3}+R_{2}(R_{1}+R_{3}+R_{4})\right)}{-R_{1}R_{3}-R_{3}R_{4}-R_{2}(R_{1}+R_{3}+R_{4})-R_{5}(R_{1}+R_{3}+R_{4})}\Delta I^{trans}$$

The transepithelial potential difference can now be calculated by:

*V^trans^* = *I*_1_*R*_1_ + *I*_2_*R*_2_ (= *I*_4_*R*_4_ + *I*_5_*R*_5_) (19)

Fluxes directed from lumen to cell and to *lis*, from cell to peritubular bath and to *lis*, and from *lis* to peritubular bath have a positive sign. Electric potentials are referenced with respect to the peritubular (serosal) side, i.e., *ψ^ps^* ≡ 0.

### 3.7. Numerical Methods

The transport equations for water and solutes are as follows:

(20) $$\frac{d\bar{V}}{dt}=\sum_{m}J_{\bar{V}}$$

(21) $$\frac{d(\bar{V}\cdot C_{S})}{dt}=\sum_{m}J_{S}$$

$\bar{V}$ denotes the volume of the cell or the lateral intercellular space, and $J_{\bar{V}}$ and $J_{S}$ denote the water and solute fluxes through the various membranes, respectively, where *m* indicates the membrane (*m* = 1–5). The left-hand sides are zero in a steady state, while the study of time-dependent behavior requires Equations (20) and (21) to be simulated. To solve the equations in time, we apply second-order accurate, three-point backward difference schemes (Taylor expansion) as follows:

(22) $$\frac{1}{2\Delta t}\left[3{\bar{V}}^{\left(n\right)}-4{\bar{V}}^{\left(n-1\right)}+{\bar{V}}^{\left(n-2\right)}\right]=\sum_{j}J_{\bar{V}}^{\left(n\right)}$$

(23) $$\frac{1}{2\Delta t}\left[3{\left(\bar{V}\cdot C_{S}\right)}^{\left(n\right)}-4{\left(\bar{V}\cdot C_{S}\right)}^{\left(n-1\right)}+{\left(\bar{V}\cdot C_{S}\right)}^{\left(n-2\right)}\right]=\sum_{m}J_{S}^{\left(n\right)}$$

The index n refers to time *t^n^*, and Δ*t* is the time step, such that *t^n^* = *t^n^*^−1^ + Δ*t*. Thus, the equations are solved for all variables with index n at time *t* = *t^n^*, leaving the remaining terms known from former time steps. The equations are solved together with the above equations for electroneutrality and the compliance model. The strongly coupled nonlinear equations were solved for machine accuracy through a conventional iterative Newton–Raphson method. In forming the Jacobian matrix, rather than analytical differentiations, we employed a simple difference scheme. The term ‘sampling frequency’ used in analyses of non-steady-state behavior refers to the chosen frequency of time steps [5].

### 3.8. Independent Variables

Appendix B lists the independent variables of the model selected for simulating proximal tubule S3 segments [58,59]. For model computations, we apply MKSA units for physical constants, independent variables, and computed dependent variables; however, in the text, they are converted to the units generally used in the physiological literature. The solute permeabilities were chosen for obtaining values within the physiological range of intracellular ion concentrations, transcellular fluxes and membrane potentials [58], while the hydraulic conductances were obtained from refs. [60,61,62].

## 4. Results

In calibrating the model for kidney proximal tubules, it became clear that published intracellular cation concentrations cover unusual ranges, as exemplified by generally very low intracellular potassium concentrations, e.g., 79 mM (*Necturus* [63]), 68 mM (rabbit [64]), and 113 mM (rat [65]). Likewise, available measurements indicated the intracellular sodium concentration of proximal tubule cells to be in the high end compared to other cell types, e.g., 44 mM (rabbit [66]) and 17.5 mM (rat [67]). Proximal tubules are metabolically very active due to the reabsorption of electrolytes, glucose, and other nutrients from the glomerular filtrate, in humans of 180 L per day, which places the kidney as second only to the heart with respect to specific metabolic rate [68]. Here, we analyze the mechanisms by which the tightly regulated cell sodium and potassium concentrations under physiological conditions can deviate so significantly under experimental conditions. As argued below, we suggest that the electromotive force of the sodium pump is of significance for the deviating cation concentrations, thus hinting at insufficient availability of free oxygen. Table 1 covers a range of electromotive forces of the sodium pump *E^pump^* (Equation (5a)), here indicated by the reversal potential of the sodium pump current, $V_{rev}^{pump}$ (see Section 3) and the associated free energy change in ATP hydrolysis, $\Delta G_{ATP}$ , in the vicinity of the pump’s ATPase site. The reference concentrations indicated in line 1 are about those of cardiac muscle cells of specific energy metabolism comparable to that of the proximal tubule [69]. The standard $\Delta G_{ATP}$ of cytosolic ATP hydrolysis (column 2) agrees well with measured values, varying little between brain, liver and muscle cells [45]. As can be seen, our computations indicate that both $C_{Na}^{c}$ and $C_{K}^{c}$ dramatically depend on *E^pump^*, which provides a plausible biophysical explanation for measured intracellular cation concentrations of proximal tubules deviating from those of other cell types. This is unlike the two kinetic variables, the sodium pump flux (column 9) and the volume flow (column 10), which are related to the number and turnover of functionally expressed pumps, both of which are weakly dependent on the *E^pump^*. Given that, these last-mentioned variables are indicators of well-functioning in vitro preparations; this is the other remarkable result of Table 1. The insignificant change in transepithelial potential difference, *V^trans^*, with *E^pump^* (column 8) is due to its ohmic relationship with the active Na current and the leak current carried predominantly by junctional claudin-17/10a, largely having invariable conductance at the prevailing very small transepithelial potential difference. The electrochemical work performed by the kidney proximal tubule is fueled by lipid oxidation, which requires relatively more O_2_ than glucose metabolism; e.g., palmitic acid yields 106 ATP per 23 O_2_, whereas glucose oxidation yields 38 ATP per 6 O_2_ [70]. With compromised in vitro O_2_ delivery to tubule cells, these cells inevitably enter partial anaerobic metabolism, which in turn leads to a reduced ATP/ADP ratio with associated reduced $\Delta G_{ATP}$ and *E^pump^*. In the laboratory, this would be reflected in the reversal potential of the pump current, with $V_{rev}^{pump}$ being numerically smaller than its physiological value of about −200 mV. Free protons generated by mitochondrial metabolism have to be eliminated from the cells. It is likely that the highly expressed Na/H exchanger of isolated proximal tubules [14,71] serves this function.

### 4.1. The Steady State with No Ion Recirculation

**Figure 2:**
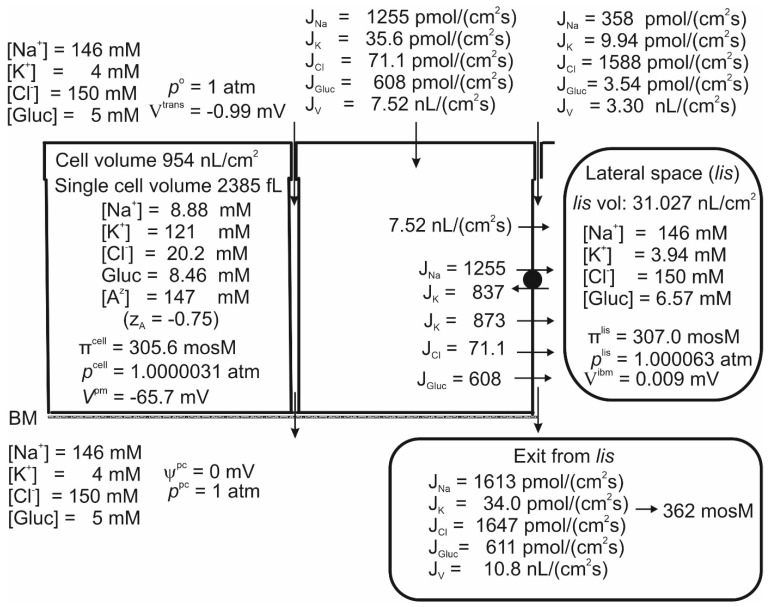
Computations predict that mammalian kidney late proximal straight tubules spontaneously generate a hyperosmotic transportate. Model epithelium ‘perfused on both sides’ with simulated saline containing (mM), 146 Na, 4 K, 150 Cl and 5 glucose, as indicated on the left-hand top and left-hand bottom panels, respectively. Notably, the epithelium generates significant transepithelial water flow at transepithelial osmotic equilibrium. Note that the space between the cells, *lis*, is slightly hyperosmotic (307 mosM) relative to bilateral bathing solutions of similar composition (305 mosM), while the fluid flowing into the peritubular space across *ibm* is significantly hyperosmotic (362 mosM).

Studies of proximal straight tubules exposed to bicarbonate-free solutions reported a small transepithelial potential difference of approximately −1 mV [72,73]. Our model tubule in bicarbonate-free saline supplied with 5 mM glucose absorbed H_2_O, Na, K, Cl and glucose at *V^trans^* = −0.99 mV, Table 1 line 1 and Figure 2. While the negative sign of *V^trans^* is a consequence of the active Na flux being conductive and inwardly directed [17,72], its numerical value is given by the active Na flux shunted by the Cl conductance mediated by claudin-10a in tight junctions [51]. Apical SGLT1 contributes with a relatively significant water flux of 4.37 nL·cm^−2^·s^−1^; that is, compared to the transepithelial water uptake of 10.8 nL·cm^−2^·s^−1^, as much as ~40% is carried by luminal SGLT1 driven by cotransport with Na and glucose. The cellular uptake of Na across the luminal membrane, $J_{Na}^{apical}$ = 1255 pmol·cm^−2^·s^−1^, occurs via three pathways: (*i*) the small flux carried by the cotransporter, $J_{Na}^{am\;CO}$ = 35.6 pmol·cm^−2^·s^−1^ (Equation (2)); (*ii*) the even smaller flux through the amiloride-inhibitable Na channel, $J_{Na}^{ENaC}$ = 3.86 pmol·cm^−2^·s^−1^ (Equation (1)), which is difficult to detect experimentally; and (*iii*) the numerically dominating SGLT1, $J_{Na}^{SGLT1}$ = 1215 pmol·cm^−2^·s^−1^ (Equation (3)). The Na/K pump energizes the low cellular Na concentration, here 8.88 mM, and the hyperosmotic *lis* of 307 mosM. The paracellular Na uptake of 358 pmol·cm^−2^·s^−1^ is driven by solvent drag via claudin-2 water permeable cation selective channels of the tight junction [52], indicating that a significant fraction of transepithelial Na uptake bypasses the Na/K pump. This finding is compatible with the early study of Na reabsorption by the mammalian kidney in vivo, which concluded that the reabsorption of tubular fluid is significantly more efficient energetically than estimated from the Na-flux dependent O_2_ consumption, which is a measure of ATP hydrolysis at the lateral Na/K-pump ATPase [74,75]. The uptake of K via the luminal cotransporter and by solvent drag in tight junction claudin-2-mediated channels results in a K concentration in the absorbate of *C_K_* = (35.6 + 9.94)/10.8 pmol·cm^−2^·s^−1^·nL^−1^·cm^2^·s^1^ = 4.22 mM (Figure 2), which is in the range of K concentrations in the extracellular fluid [76]. As shown in Figure 2, the paracellular K uptake did not exceed 9.74/10.9 pmol·cm^−2^·s^−1^·nL^−1^·cm^2^·s = 0.91 mM. Therefore, the apical cotransporter effectively compensates for the component driven by solvent drag via the paracellular route, which is insufficient for reabsorbing potassium. At a cell potential of −65.7 mV and an intracellular Cl concentration of 20.2 mM (Figure 2), the Cl distribution between the cell and the symmetrical bathing solutions is above thermodynamic equilibrium (*E_Cl_* = −53.5 mV). By being in series with an apical Na-gradient-driven cotransporter [24], here assumed to be a 1Na–1K–2Cl mechanism [23,24,26], the lateral Na/K pump also energizes this steady-state disequilibrium.

### 4.2. Crosstalk between Electrogenic SGLT1 and Electrogenic Lateral Na/K Pumps

**Figure 3:**
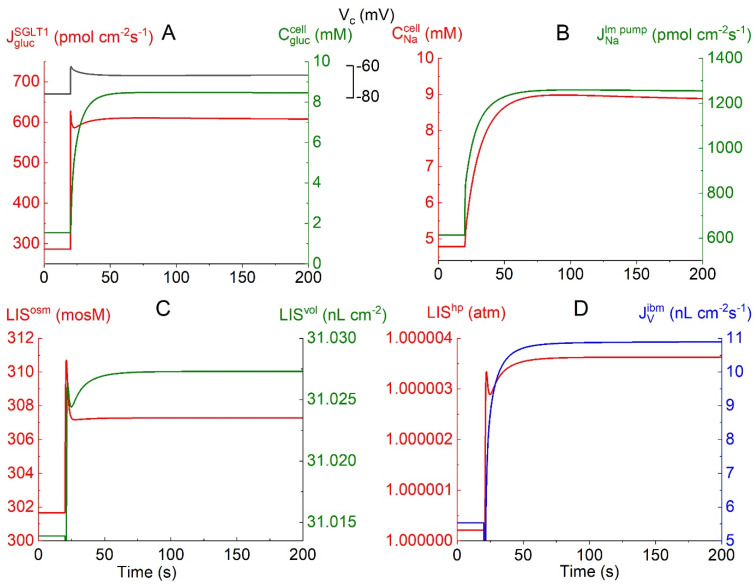
Computed trans- and intraepithelial biophysical variables following a step change in bilateral glucose from 0.5 to 5 mM at *t* = 20 s. (**A**) The evoked fast increase in rheogenic SGLT1 flux passing a peak of 628 pmol·cm^−2^·s^−1^ (red) leads to fast cell depolarization from −77.1 to −60.7 mV (black). The subsequently slower relaxation to −65.7 mV follows the transient decrease in rheogenic 2Na–1glucose uptake, while the cellular glucose concentration increases to 8 mM (green). (**B**) The fast increase in the depolarization-generated rheogenic active Na flux across *lm* proceeds through a slower increase caused by the increase in the cellular Na concentration, as indicated in the red graph. (**C**) Uptake by *lis* of NaCl results in an increase in the osmolarity (LIS^osm^) and volume (LIS^vol^) of the lateral intercellular space. (**D**) The increase in *lis* volume results in an increase in hydrostatic pressure (LIS^hp^), which drives fluid out of *lis* into the peritubular space, resulting in a steady state transepithelial water uptake of 10.8 nL·cm^−2^·s^−1^ (blue).

Figure 3A–D depicts time-dependent changes in selected physiological variables in response to a bilateral monoexponential (τ = 1 s) 10-fold increase in external glucose concentrations from 0.5 to 5 mM. Figure 3A: The flux of glucose carried by the voltage-dependent SGLT1, $J_{Gluc}^{SGLT1}$ = 286 pmol·cm^−2^·s^−1^, at $C_{Gluc}^{o}$ = 0.5 mM prior to the bilateral glucose step, increases to a peak value of 628 pmol·cm^−2^·s^−1^. Simultaneously, the apical membrane depolarizes from −77.1 to −61.0 mV due to the ohmic membrane potential response to the fast inflow of two positively charged sodium ions per glucose molecule. The subsequent decrease in $J_{Gluc}^{SGLT1}$ to 608 pmol·cm^−2^·s^−1^ and increase in *V*^cell^ to −65.8 mV are caused by the following entangled interactions. The fast cellular uptake of glucose causes $C_{Gluc}^{c}$ to increase steeply from 1.54 to 8.45 mM. Therefore, the glucose influx and associated ohmic membrane potential response diminish *pari passu* with a decreasing glucose concentration difference between the cell and the luminal solution. Figure 3B: The stimulated $J_{Gluc}^{SGLT1}$ causes $C_{Na}^{c}$ to increase from 4.8 to 8.9 mM. The flux of sodium ions pumped into the lateral intercellular space, $J_{Na}^{lm,pump}$ , increases from 613 to 1255 pmol·cm^−2^·s^−1^, initially with a fast time course because of the exponential SGLT1-generated cell depolarization, and subsequently slowly owing to the similarly slow increase in $C_{Na}^{c}$ . Figure 3C: The *lis* uptake of Na and Cl leads to an increase in the *lis* osmolarity from 301.6 mosM through a transient peak at 310.7 mosM before attaining a steady-state value of 307.3 mosM, associated with solute fluxes across the interspace basement membrane into the peritubular space. This in turn drives the volume expansion of the *lis* from 31.014 to 31.027 nL·cm^−2^. The water uptake from luminal fluid occurs through three different pathways: via AQP1 by osmosis, via SGLT1 associated with glucose influx, and by flow in the water-permeable claudin-2 of tight junctions, as analyzed below. Figure 3D: The associated increase in the hydrostatic pressure of *lis* constitutes the force driving water across the interspace basement membrane (*ibm*) with a low reflection coefficient into the peritubular compartment, at 200 s $J_{V}^{SGLT1}$ = 10.8 nL·cm^−2^·s^−1^. Taken together, the results shown in Figure 3A–D indicate instantaneous ‘crosstalk’ between apical rheogenic SGLT1 fluxes and the lateral Na/K pump flux by ~1:1 electric coupling between apical and lateral membrane depolarizations owing to the relatively very small junctional resistance.

### 4.3. Contributions of Epithelial Water Pathways to Transepithelial Water Absorption

**Figure 4:**
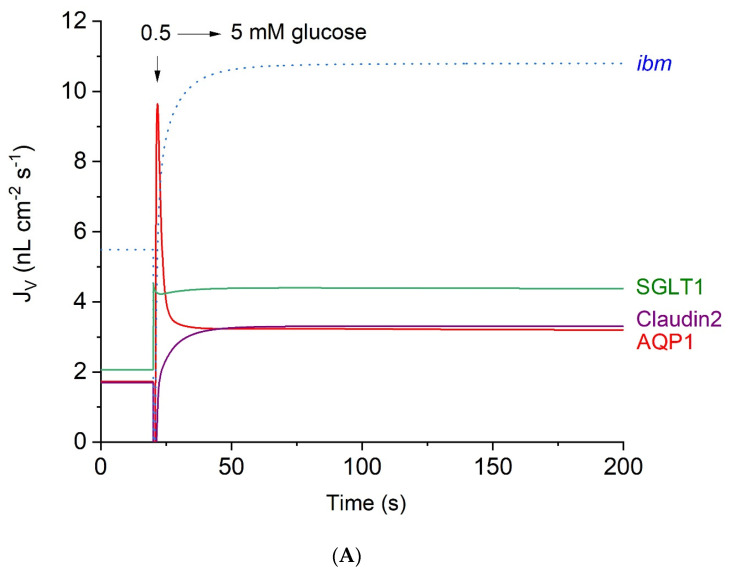

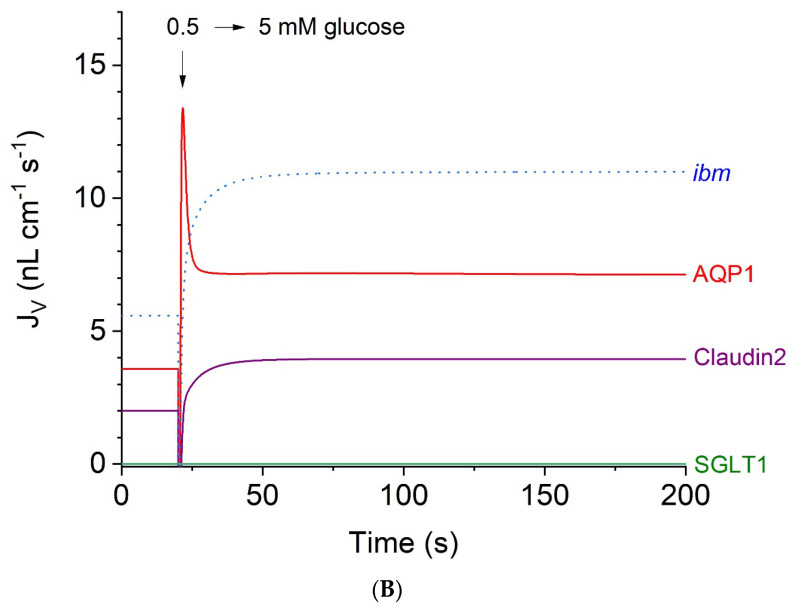
(**A**) Computed water flows in the three entrance pathways of the proximal tubule S3 segment, continued from Figure 3A–D. At 20 s, the glucose concentration increased bilaterally from 0.5 to 5 mM. The time course of *inward* water transport through two pathways in the apical membrane, AQP1 and SGLT1, was compared with that of junctional water transport through cation-permeable claudin-2. The dotted blue graph shows the time course of fluid *exit* across the interspace basement membrane (*ibm*) approaching a steady-state flow of 10.8 nL·cm^−2^·s^−1^. This is the sum of the stationary flows in the three luminal entrance pathways, AQP = 3.20 nL·cm^−2^·s^−1^, claudin-2 = 3.30 nL·cm^−2^·s^−1^, and SGLT1 = 4.32 nL·cm^−2^·s^−1^. (**B**) Computed water flows in the entrance pathways of the proximal tubule S3 segment, continued from (**A**). The protocol of (**A**) was applied, however, eliminating the water channel in SGLT1 (green graph) by replacing Equation (3b) in Section 3 with $J_{H_{2}O}^{SGLT1}=0\times J_{Gluc}^{SGLT1}$ . The time course of inward water transport through the apical membrane, AQP1 and SGLT1, respectively, is compared to that of the junctional water transport through cation-permeable claudin-2. The dotted blue graph shows the time course of fluid exit across the interspace basement membrane (*ibm*) approaching a steady-state flow of 11.0 nL·cm^−2^·s^−1^, which is the sum of stationary flows in the three luminal entrance pathways, AQP = 7.07 nL·cm^−2^·s^−1^, claudin-2 = 3.93 nL·cm^−2^·s^−1^, and SGLT1 = 0 nL·cm^−2^·s^−1^.

In our mathematical treatment, water flow across the tubular epithelium is separated into three entrance pathways of quite different natures; see Figure 4A, which continues Figure 3A–D. (*i*) The classical aquaporin mechanism AQP1 (Equation (3a)) carries no more than 34.6% of the total water uptake of 10.8 nL·cm^−2^·s^−1^. (*ii*) Water transport in SGLT1 coupled to the inward fluxes of Na and glucose amounts to a steady state flow of 4.38 nL·cm^−2^·s^−1^; and (*iii*) water transport in junctional claudin-2, which is also permeable to small cations (Equation (9a)), carries a stationary water flow of 3.30 nL·cm^−2^·s^−1^. In similar computations shown in Figure 4B, we ‘removed’ the water pathway in the SGLT1 transporter. The remarkable result is that the stationary water flow generated by AQP1 doubled to 7.12 nL·cm^−2^·s^−1^, thus compensating for the eliminated water flow that was mediated by SGLT1, i.e., 10.8 nL·cm^−2^·s^−1^ in Figure 4A versus 11.0 nL·cm^−2^·s^−1^ in Figure 4B. To comprehend this result, we recall that the computations were carried out at transepithelial thermodynamic equilibrium, implying that the rate of water uptake depends on the active transepithelial Na flux, which is the same, 1255 pmol·cm^−2^·s^−1^, in the two situations.

### 4.4. Isosmotic Transport

**Figure 5:**
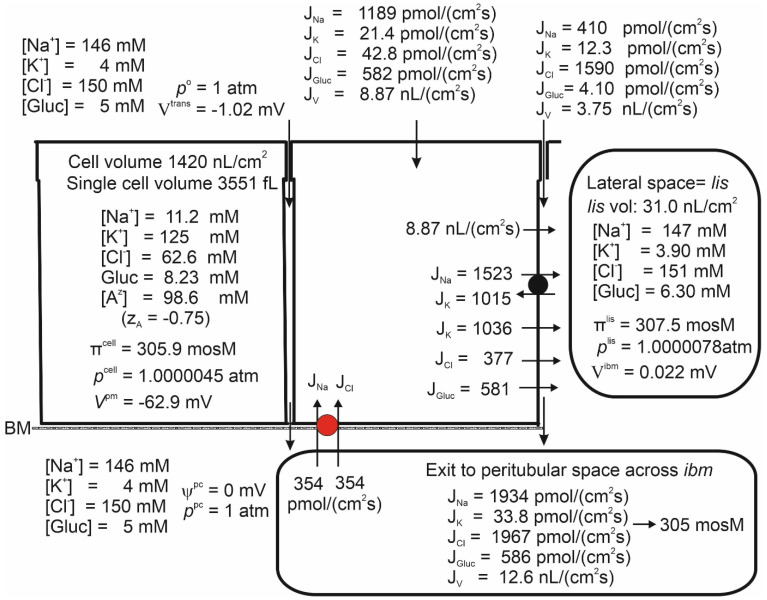
Isosmotic transport was achieved by regulating the recirculation flux of small ions between the lateral intercellular space and the peritubular solution; see Figure 1B. As in the small intestine, isosmotic transport occurs through regulation of the activity of the cotransporter of the peritubular membrane (red). Notably, significant cell swelling from 966 (Figure 2) to 1420 nL/cm^2^ (above) was caused by the uptake of Na and Cl via the cotransporter. The water flows in the two apical pathways are as follows. AQP-mediated 4.52 nL·cm^−2^·s^−1^ and SGLT1-mediated 4.35 nL·cm^−2^·s^−1^.

The fluid reabsorbed by the proximal tubule is in near-thermodynamic equilibrium with extracellular fluid [8,77,78,79,80]. The general and important finding of Figure 2 is that the fluid transported from the tubular lumen to the peritubular space is significantly hyperosmotic to bathing solutions. Inspection revealed that the fluid emerging from *lis* has greater osmolarity than the fluid of departure in casu 366 versus 307 mosM, which is qualitatively similar to our analysis of proximal convoluted tubules [5] and compatible with the Diamond–Bossert analysis of the gallbladder [81]. This indicates that diffusion rather than convection (solvent drag) dominates mass movement from the lateral intercellular space to the peritubular compartment, effectively preventing absorbed fluid from becoming isosmotic to body fluids. Faced with a similar challenge in studies of the vertebrate small intestine, Hans Ussing suggested that a way of securing isosmotic transport would be to recirculate the ‘surplus of ions’ back into the lateral intercellular space. Aspects of the Na recirculation theory were investigated for fluid absorption in the small intestine with bumetanide-inhibitable 1Na–1K–2Cl cotransporters in the contra-luminal cell membrane [82,83] and for the exocrine glands of frog skin by whole-cell and single-channel patch-clamp studies, indicating the expression of Na channels in the luminal membrane of this secretory epithelium [84,85]. In the present study, we investigated the effect of Na recirculation by incorporating a Na–Cl cotransporter into the peritubular membrane, similar to our previous study of proximal tubules [5,6]. We approached this problem by starting from the already obtained mathematical solution with no recirculation (Figure 2). Then, the rate of ion recirculation was increased iteratively while checking for osmolarity of the transported fluid. Figure 5 shows that this procedure caused the absorbed fluid to converge to osmotic equilibrium with the peritubular fluid. The principal finding is that, relative to the active Na flux, the Na recirculation flux of the S3 segment, 359/1543 = 0.23, is significantly smaller than that of the intestinal preparation of 0.65 ± 0.03, which has been studied in the amphibian small intestinal epithelium [82]. The logical explanation for this difference is that apical AQP1 constitutes a significant apical water pathway of the S3 segment, while the small intestine is relatively ‘watertight’ [29,86] because of a lack of aquaporin gene expression in fluid-absorbing enterocytes [28].

### 4.5. Isosmotic Transport Requires Cell Volume Regulation to Maintain Cellular Homeostasis

**Figure 6:**
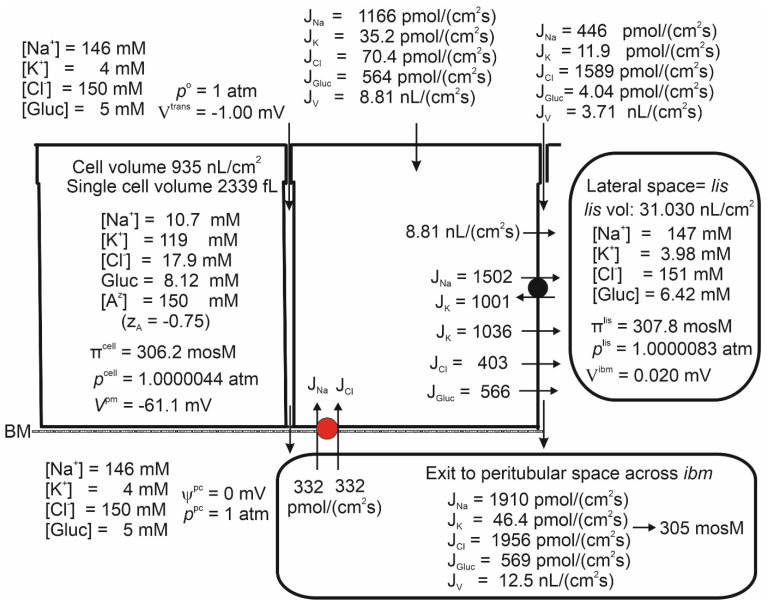
Increasing the lateral membrane’s Cl permeability from 0.3 × 10^−8^ m/s (Figure 5) to 0.6 × 10^−7^ m/s to simulate VRAC activation decreased the epithelial cell volume back to its ‘physiological value’ as in Figure 2. The water flow mediated by AQP was 4.73 nL·cm^−2^·s^−1^, which is about the same as the water flow mediated by SGLT1, 4.07 nL·cm^−2^·s^−1^. The water uptake by solvent drag through lateral intercellular space was 3.71 nL·cm^−2^·s^−1^.

By introducing Na recirculation to achieve isosmotic transport, $C_{Cl}^{c}$ increased from 20.2 to 62.6 mM with an associated cell volume expansion of Δ*Vol* = 1420 − 954 = 466 nL/cm^2^ (Figure 2 and Figure 5). Following osmotic swelling in vitro, the cell volume of the proximal straight tubule rapidly decreased toward its original volume prior to osmotic challenge [87], which is now known to be accomplished by stimulation of the volume-regulated anion channel VRAC [88]. If the cell volume of the model epithelium is forced back toward its control volume (Figure 2) by increasing $P_{Cl}^{lm}$ (‘VRAC activation’), we obtain a new set of dependent variables that still govern isosmotic transport (Figure 6). As shown in Figure 5 and Figure 6, the volume decrease is accompanied by a loss of cell K of 125·1420–120·956 = 66,235 pmol·cm^−2^. It is reassuring for our modelling that this loss of intracellular potassium occurred in response to the VRAC stimulation of osmotically swelled perfused straight tubules [89].

## 5. Discussion

Renal fluxes of ions, glucose, and water constitute a mutually interdependent functional network that is impossible to comprehend intuitively. In the present and previous studies [5], we delineated the interdependence of intraepithelial ion and water fluxes and their driving forces along the proximal tubule. The flux equation of SGLT1 comprises the dependence on apical membrane potential together with the experimentally described saturation for both substrates by a mechanism similar to that of the Na uptake by frog skin (Equation (3b)) [16]. Furthermore, we incorporated water fluxes in SGLT1 coupled stoichiometrically to the solute fluxes according to experiments [2,29], 1 glucose: 2 Na: 400 H_2_O. The frictional force driving glucose through the water-permeable pore of the tight junction into the peritubular space has not been previously considered. By incorporating this mechanism into our model, we are able to discuss the quantitative significance of paracellular glucose fluxes for overall glucose recovery along the proximal tubule.

In evaluating analytical computations and the conclusions drawn from these, we emphasize that although the applied mathematical equations conform to classical concepts in biophysics, the quantitative outcome of the modeling depends on the values of independent variables. With some of the independent variables impossible to obtain experimentally and others unavailable from common preparations, we selected the independent variables to reproduce a range of concentrations, membrane potentials and fluxes in agreement with experiments on mammals, including humans. In our calibration of the model for reproducing published observations, the large range of measured intracellular cation concentrations surprised us. This forced us to perform a quantitative biophysical analysis, which suggested that insufficiently oxygenated preparations caused the high intracellular Na and the generally very low intracellular K concentrations reported for in vitro preparations. In the present study, these measurements were disregarded in our calibration of the model. This procedure is trivial and not controversial.

The function of the straight tubule was studied under conditions similar to those of the kidney in situ, which is defined by similar composition of the solutions bathing the two sides of the tubular epithelium and an insignificant transepithelial electrical potential difference. This means that solute reabsorption took place in the absence of an external driving force for transepithelial water flow. Therefore, the water uptake was driven by metabolic energy mediated by the Na/K pump in the lateral membrane of the tubular epithelial cells. This fundamental feature of the kidney proximal tubule was revealed by confirming that the transepithelial water uptake at a constant active Na uptake is independent of the presence of solute-coupled water flow in the Na–glucose cotransporter, SGLT1.

By applying our previously derived mathematical equations governing paracellular solvent drag [49] to the recently identified sodium- and water-permeable junctional claudin-2 channels [51,52], we solved the early enigma that a significant fraction of the transepithelial Na reabsorption by mammalian kidney bypasses the sodium pump [74,75].

Another issue that demands attention concerns isosmotic fluid absorption. The underlying mechanism has been debated ever since it was realized that the osmolarity of luminal fluid does not change measurably during the absorption of ~70% of the volume generated by glomerular filtration [8,77,78,79,90]. In textbooks [76,80], isosmotic transport is assumed to be an inevitable condition owing to the documented high hydraulic cell membrane conductances of kidney epithelia. Our quantitative analysis of proximal tubules revealed that significant diffusion permeabilities of an interspace basement membrane with a low reflection coefficient result in the reabsorbed fluid being hyperosmotic to body fluids. Thus, isosmotic transport is not a simple consequence of passive ion fluxes across highly water-permeable membranes, but requires tightly regulated reabsorption of ions depending on metabolic energy. In our treatment, the osmolarity of the transported fluid is a derived quantity with isosmotic transport resulting from regulation of the recirculation of small diffusible ions depending on additional activity of the lateral Na/K pump in accordance with experimental studies on other transporting epithelia [82,84].

In the above analyses, we also focused on the interdependence of the glucose flux carried by SGLT1 in the luminal membrane and the transepithelial water flow. In summarizing the results regarding fluid absorption by late proximal straight tubules, we could conclude that electric crosstalk between rheogenic apical SGLT1 and rheogenic depolarization-activated lateral Na/K pumps constitutes both a logical and a sufficient explanation for the observed coupling between SGLT1-mediated glucose reabsorption and transepithelial water uptake driven by the increased osmolarity of *lis*. The functional linkage between apical and lateral rheogenic fluxes is particularly tight in the kidney proximal tubule owing to the large paracellular electrical conductance, which ensures near 1:1 coupling between luminal and lateral membrane depolarizations.

## Figures and Tables

**Table 1:** Biophysical model analysis of the influence of the electromotive force of the sodium pump (*E^pump^* of Equation (5a)) on proximal tubule function. *E^pump^* is a measure of the free energy change in ATP hydrolysis in the vicinity of pump sites and may be experimentally obtained by measuring the reversal potential of the pump current (column 1) with a physiological value of −200 mV, corresponding to 1/3 of the prevailing ${\Delta G}_{ATP}$ of −58 kJ/mole (column 2, line 1). Generally, $V_{rev}^{pump}$ ( $\Delta G_{ATP}$ ) provides information on the availability of oxygen for oxidative phosphorylation during the experiment. Cation concentrations of the peritubular solution were maintained at $C_{Na}^{ps}$ = 140 mM and $C_{K}^{ps}$ = 4 mM. Columns 3 ( $C_{Na}^{c}$ ) and 4 ( $C_{K}^{c}$ ) cover the large range of intracellular cation concentrations measured over time in studies of the kidney proximal tubule.

$V_{rev}^{pump}$	$\Delta G_{ATP}$	$C_{Na}^{c}$	$C_{K}^{c}$	*V^c^*	$E_{Na}-V^{c}$	$E_{K}-V^{c}$	*V^trans^*	$J_{Na}^{pump}$	$J_{V}^{ibm}$
mV	kJ/mol	mM	mM	mV	mV	mV	mV	pmol·cm^−2^·s^−1^	nL·cm^−2^·s^−1^
−200	−58.0	8.77	122	−65.5	140	−25.4	−0.99	1250	10.8
−180	−52.2	10.7	120	−65.1	135	−25.4	−0.98	1243	10.8
−160	−46.4	13.9	117	−64.4	127	−25.4	−0.97	1230	10.6
−140	−40.6	20.4	110	−63.0	116	−25.3	−0.95	1204	10.4
−120	−35.2	35.1	95.8	−59.5	97.5	−25.3	−0.90	1138	9.85
−100	−29.0	60.2	71.6	−52.3	75.9	−24.7	−0.79	1004	8.69

## Data Availability

All required data are given explicitly in the paper.

## Appendix A. Definitions and Symbols of Variables and Constants

Concentration of *j* (*Na*, *K*, *Cl*, *A*, *or glucose*) in compartment *comp* (*o*, *cell*, *lis or ps*)	$C_{j}^{comp}$
Osmolarity of compartment indicated by superscript (*o*, *cell*, *lis or ps*)	$\pi^{comp}$
Hydrostatic pressure of compartment indicated (*o*, *cell*, *lis or ps*)	*p^comp^*
Electrical potential of compartment indicated (*o*, *cell*, *lis or ps*) with $\psi^{ps}\equiv 0$	$\psi^{comp}$
Transepithelial potential difference, $\psi^{o}-\psi^{ps}$	*V^trans^*
Electrical potential difference between *lumen* and *cell*, $\psi^{o}-\psi^{c}$	*V^am^*
Electrical potential difference between *cell* and *lis*, $\psi^{c}-\psi^{lis}$	*V^lm^*
Electrical potential difference between *cell* and *peritubular space*, $\psi^{c}-\psi^{ps}$	*V^ps^*
Passive permeability of *j* in membrane *m* (*am*, *lm*, *pm*, *tj*, *ibm*)	$P_{j}^{m}$
Reflection coefficient of *j* (Na, K, Cl, glucose) in *m* (*tj*, *ibm*)	$\sigma_{j}^{m}$
Flux of *j* (= Na, K, Cl, glucose) across *m* (*am*, *lm*, *pm*, *tj*, *ibm*)	$J_{j}^{m}$
Electrical current carried by *j* (Na, K, Cl) across *m* (*am*, *lm*, *pm*, *tj*, *ibm*)	$I_{j}^{m}$
Integral ion (*j*) conductance of membrane *m* (*am*, *lm*, *pm*, *tj*, *ibm*)	$G_{j}^{m}$
Water volume flux across *m* (*am*, *lm*, *pm*, *tj*, *ibm*)	$J_{V}^{m}$
Hydraulic conductance of membrane *m* (*am*, *lm*, *pm*, *tj*, *ibm*)	$L^{m}$
Osmotic permeability of *m* (*am*, *lm*, *pm*, *tj*, *ibm*)	$P_{f}^{m}$
Relative compliance constant of membrane *m* (*am*, *lm*, *pm*)	$\mu^{m}$
Absolute compliance constant of *lm*	${\bar{\mu }}^{lm}$
Empirical constant of 1Na–1K–2Cl cotransporter of membrane *m*	$K^{NaK2Cl,m}$
Apparent dissociation constants of K binding of Na/K pump at *lm*	$K_{K}^{pump,lm}$
Empirical apparent dissociation constant of Na–glucose transporter at *am*	$K_{Gluc}^{Gluc\cdot Na}$
Empirical apparent dissociation constant for glucose of Na–glucose transporter at *am*	$K_{Na}^{Gluc\cdot Na}$
Maximum turnover of glucose exchanger at *lm*	$J_{Gluc}^{\max,lm}$
App dissociation const. of symmetrical carrier at *lm*	$K_{Gluc}^{lm}$
Turnover constant of 2 Na–1 glucose transporter at *am*	$P^{Gluc\cdot Na}$
Temperature in K	*T*
Faraday	*F*
Universal gas constant	*R*

## Appendix B. Independent Variables

*o*: outer (luminal) compartment, *ps*: peritubular compartment, *am*: apical membrane; *lm*: lateral membrane; *pm*: peritubular membrane; *tj*: tight junction; *ibm*: interspace basement membrane. The listed numbers refer to the model’s standard version.

**Name**	**Symbol**	**Value**	**MKSA-Unit**
Hydraulic conductance of *am*	*L^am^*	2.125·10^−11^	m^3^·s^−1^·N^−1^
Hydraulic conductance of *pm*	*L^pm^*	3.50·10^−16^	m^3^·s^−1^·N^−1^
Hydraulic conductance of *lm*	*L^lm^*	2.125·10^−11^	m^3^·s^−1^·N^−1^
Hydraulic conductance of *tj*	*L^tj^*	9.00·10^−12^	m^3^·s^−1^·N^−1^
Hydraulic conductance of *ibm*	*L^ibm^*	2.31·10^−07^	m^3^·s^−1^·N^−1^
Na concentration in outside (luminal) compartment	$C_{Na}^{o}$	146	mol·m^−3^
K concentration in outside (luminal) compartment	$C_{K}^{o}$	4	mol·m^−3^
Cl concentration in outside (luminal) compartment	$C_{Cl}^{o}$	150	mol·m^−3^
Glucose concentration in luminal compartment Vary in Table	$C_{Gluc}^{o}$	5	mol·m^−3^
Na concentration in peritubular (inside) compartment	$C_{Na}^{ps}$	146	mol·m^−3^
K concentration in peritubular (inside) compartment	$C_{K}^{ps}$	4	mol·m^−3^
Cl concentration in peritubular (inside) compartment	$C_{Cl}^{pc}$	150	mol·m^−3^
Na GHK permeability of *am*	$P_{Na}^{am}$	1.00·10^−10^	m·s^−1^
K GHK permeability of *am*	$P_{K}^{am}$	1.00·10^−14^	m·s^−1^
Cl GHK permeability of *am*	$P_{Cl}^{am}$	1.00·10^−14^	m·s^−1^
Na GHK permeability of *pm*	$P_{Na}^{pm}$	1.00·10^−13^	m·s^−1^
K GHK permeability of *pm*	$P_{K}^{pm}$	1.00·10^−13^	m·s^−1^
Cl GHK permeability of *pm*	$P_{Cl}^{pm}$	1.00·10^−13^	m·s^−1^
Na GHK permeability of *lm*	$P_{Na}^{lm}$	3.00·10^−15^	m·s^−1^
K GHK permeability of *lm*	$P_{K}^{lm}$	5.00·10^−7^	m·s^−1^
Cl GHK permeability of *lm*	$P_{Cl}^{lm}$ Figure 6	3.00·10^−8^ 6.00·10^−7^	m·s^−1^ m·s^−1^
Na GHK permeability of *tj* (Equation (6))	$P_{Na}^{tj}$	1.50·10^−7^	m·s^−1^
K GHK permeability of *tj* (Equation (6))	$P_{K}^{tj}$	2.205·10^−7^	m·s^−1^
Cl GHK permeability of *tj* (Equation (1))	$P_{Cl}^{tj}$	2.70·10^−6^	m·s^−1^
Glucose permeability of *tj* (Equation (7a))	$P_{Gluc}^{tj}$	5.00·10^−10^	m·s^−1^
Na GHK permeability of *ibm* (Equation (6))	$P_{Na}^{ibm}$	1.20·10^−5^	m·s^−1^
K GHK permeability of *ibm* (Equation (6))	$P_{K}^{ibm}$	1.76·10^−6^	m·s^−1^
Cl GHK permeability of *ibm* (Equation (6))	$P_{Cl}^{ibm}$	3.50·10^−5^	m·s^−1^
Glucose permeability if *ibm* (Equation (7b))	$P_{Gluc}^{ibm}$	3.50·10^−6^	m·s^−1^
1Na–1K–2Cl cotransporter constant of *am*	$K^{NaK2Cl,am}$	0.28·10^−14^	m^10^·s^−1^·mol^−3^
1Na–1Cl cotransporter constant of *pm*	$K^{NaCl,pm}$	dependent	m^10^·s^−1^·mol^−3^
Turnover constant of lateral 3Na/2K pump	$P_{Na,K}^{lm,pump}$	3.50·10^−4^	mol·m^−2^·s^−1^
Reversal potential of lateral 3Na/2K pump	*E^pump^*	−0.200	volt
Apparent dissociation constants of Na binding	$K_{Na}^{pump,lm}$	3.40	mol·m^−3^
Apparent dissociation constants of K binding	$K_{K}^{pump,lm}$	0.75	mol·m^−3^
Turnover constant of SGLT1 at *am*	$P^{SGLT1,\max}$	7.0·10^−13^	mol·m^−2^·s^−1^
Apparent dissociation constant for glucose at SGLT1	$K^{SGLT1}$	1.0	mol·m^−3^
Maximum turnover of glucose exchanger at *lm*	$J_{Gluc}^{\max,lm}$	3.0·10^−10^	mol·m^−2^·s^−1^
App dissociation const. of symmetry carrier at *lm*	$K_{Glu}^{lm}$	50	mol·m^−3^
Hydrostatic pressure of outer (luminal) compartment	*p^o^*	1.01325 10^5^	Pa
Hydrostatic pressure of peritubular compartment	*p^ps^*	1.01325 10^5^	Pa
Mean valence of nondiffusible intracellular anions	*z_A_*	−0.75	
Na reflection coefficient of *tj*	$\sigma_{Na}^{tj}$	0.70	
K reflection coefficient of *tj*	$\sigma_{K}^{tj}$	0.70	
Cl reflection coefficient of *tj*	$\sigma_{Cl}^{tj}$	0.45	
Glucose reflection coefficient of *tj*	$\sigma_{Gluc}^{tj}$	0.80	
Na reflection coefficient of *ibm*	$\sigma_{Na}^{ibm}$	0.0001	
K reflection coefficient of *ibm*	$\sigma_{K}^{ibm}$	0.0001	
Cl reflection coefficient of *ibm*	$\sigma_{Cl}^{ibm}$	0.0001	
Glucose reflection coefficient of *ibm*	$\sigma_{Gluc}^{ibm}$	0.0001	
Temperature	T	310	K
Faraday	*F*	96,485	C·mol^−1^
Absolute compliance constant of *lm*	$\mu^{lm}$	2.50·10^−3^	Pa^−1^
Reference volume of *lis*	*Vol* ^lis,ref^	3.10·10^−7^	m^3^·m^−2^
Cell density	*D^c^*	4.00·10^9^	Number of cells·m^−2^
Nondiffusible anions in cell	*M_A_*	3.50·10^−13^	mol·cell^−1^
