# Correction: Larsen, E.H.; Sørensen, J.N. Biophysical Analysis of a Minimalistic Kidney Model Expressing SGLT1 Reveals Crosstalk between Luminal and Lateral Membranes and a Plausible Mechanism of Isosmotic Transport. *Biomolecules* 2024, *14*, 889

**DOI:** 10.3390/biom15010048

**Published:** 2025-01-02

**Authors:** Erik Hviid Larsen, Jens Nørkær Sørensen

**Affiliations:** 1 Department of Biology, University of Copenhagen, DK-2100 Copenhagen, Denmark; 2 Department of Wind Energy, Technical University of Denmark, DK-2800 Lyngby, Denmark; jnso@dtu.dk

The authors would like to modify Equations (5a) and (5b) in Section 3.1. *Cellular Solute Flux Equations* of the published paper [1].

The authors regret printing errors and would like to highlight that these do not occur in the FORTRAN program generating the data of Table 1 and Figures 2–6, which are correctly reproduced in the published paper.

The correct equations read:

(5a) $$\begin{array}{l} J_{Na}^{lm,pump}=P_{Na,K}^{lm,pump}{\left(\frac{C_{Na}^{c}}{K_{Na}^{lm,pump}+C_{Na}^{c}}\right)}^{3}{\left(\frac{C_{K}^{lis}}{K_{K}^{lm,pump}+C_{K}^{lis}}\right)}^{2}\left[V^{lm}+E^{pump}\right]\\ J_{K}^{lm,pump}=-\frac{2}{3}J_{Na}^{lm,pump}\\ V_{m}^{lm}=\psi^{cell}-\psi^{lis}\\ I_{Na}^{lm,pump}=\frac{F\cdot P_{Na,K}^{lm,pump}}{3}{\left(\frac{C_{Na}^{c}}{K_{Na}^{lm,pump}+C_{Na}^{c}}\right)}^{3}{\left(\frac{C_{K}^{lis}}{K_{K}^{lm,pump}+C_{K}^{lis}}\right)}^{2}\left[V^{lm}+E^{pump}\right] \end{array}$$

where *F* is the Faraday constant. Introducing the pump conductance, $G^{lm,pump}$ we have:

$$I_{Na}^{lm,pump}=G^{lm,pump}[V^{lm}+E^{pump}]$$

(5b) $$G^{lm,pump}=\frac{F\cdot P_{Na,K}^{lm,pump}}{3}{\left(\frac{C_{Na}^{c}}{K_{Na}^{lm,pump}+C_{Na}^{c}}\right)}^{3}{\left(\frac{C_{K}^{lis}}{K_{K}^{lm,pump}+C_{K}^{lis}}\right)}^{2}$$

The dimension of $P_{Na,K}^{lm,pump}$ is mol∙s^−1^∙V^−1^ and that of $G^{lm,pump}$ is Siemens, both per unit area of apical membrane.

The authors apologize for any inconvenience caused and would like to state that their scientific conclusions are unaffected. This correction was approved by the Academic Editor. The original publication has also been updated.
